# Supplementary material for: Receptor repertoires of murine follicular T helper cells reveal a high clonal overlap in separate lymph nodes in autoimmunity
Source: eLife. 2021 Aug 17;10:e70053. doi: 10.7554/eLife.70053 (PMC8370764; doi:10.7554/eLife.70053)
Supplement: Supplementary file 1. [file elife-70053-supp1.docx]

Supplementary File 1: Volumes, T cell numbers, raw reads, total and unique TCRβ sequences obtained from laser-captured GC of pln (Ag1)

| PBS  4 wk p.i. | mouse | GC | captured GC volume (x 10^7^ µm^3^) | counted and estimated number of Tfh cells  (x 10^4^) | raw reads (x10^6^) | total TCRβ sequences (x10^6^)* | unique TCRβ clonotypes | number of Tfh-clonotypes subjected to analysis  >median ** |
| --- | --- | --- | --- | --- | --- | --- | --- | --- |
|  | 1 | left | 3.97 | 3.31 | 2.1 | 0.97 | 545 | 272 |
|  |  | right | 4.38 | 3.65 | 2.0 | 0.59 | 2455 | 1226 |
|  | 2 | left | 3.73 | 3.11 | 2.2 | 0.72 | 1091 | 547 |
|  |  | right | 4.20 | 3.5 | 1.6 | 0.45 | 2427 | 1207 |
|  | 3 | left | 4.06 | 3.39 | 2.3 | 0.63 | 623 | 310 |
|  |  | right | 4.03 | 3.36 | 1.6 | 1.45 | 7329 | 3646 |
|  | mean ± SD |  | 4.06 ± 0.22 | 3.39 ± 0.18 | 1.97 ± 0.30 | 0.80 ± 0.36 | 2411.67 ± 2553 | 1201.33 ± 1270 |
| Ag1  4 wk p.i. | mouse | GC | captured GC volume (x 10^7^ µm^3^) | counted and estimated number of Tfh cells  (x 10^4^) | raw reads (x10^6^) | total TCRβ sequences (x10^6^)* | unique TCRβ clonotypes | number of Tfh-clonotypes subjected to analysis  >median ** |
|  | 1 | left | 4.68 | 3.19 | 2.1 | 0.95 | 6269 | 3096 |
|  |  | right | 4.34 | 3.77 | 2.0 | 1.42 | 1919 | 958 |
|  | 2 | left | 5.16 | 3.77 | 2.3 | 1.08 | 1503 | 747 |
|  |  | right | 3.91 | 4.12 | 2.0 | 1.16 | 4378 | 2187 |
|  | 3 | left | 4.44 | 4.1 | 1.6 | 1.00 | 1467 | 729 |
|  |  | right | 4.29 | 3.57 | 2.0 | 1.21 | 4250 | 2122 |
|  | mean ± SD |  | 4.47 ± 0.42 | 3.75 ± 0.35 | 2.0 ± 0.23 | 1.14 ± 0.17 | 3297.67 ± 1968 | 1639.83 ± 974.06 |
| Ag1  2 wk p.i. | mouse | GC | captured GC volume (x 10^7^ µm^3^) | counted and estimated number of Tfh cells  (x 10^4^) | raw reads (x10^6^) | total TCRβ sequences (x10^6^)* | unique TCRβ clonotypes | number of Tfh-clonotypes subjected to analysis  >median ** |
|  | 1 | left | 4.26 | 3.62 | 2.3 | 0.70 | 1625 | 808 |
|  |  | right | 4.51 | 3.85 | 2.0 | 1.04 | 3033 | 1514 |
|  | 2 | left | 4.63 | 3.69 | 2.2 | 1.02 | 2321 | 1159 |
|  |  | right | 4.52 | 3.63 | 2.4 | 1.16 | 1672 | 835 |
|  | 3 | left | 4.29 | 3.59 | 2.1 | 0.60 | 2851 | 1422 |
|  |  | right | 4.68 | 5.87 | 2.3 | 0.55 | 2287 | 1141 |
|  | mean ± SD |  | 4.48 ± 0.17 | 4.04 ± 0.90 | 2.22 ± 0.15 | 0.85 ± 0.26 | 2298.17 ± 581 | 1146.50 ± 290 |
| Ag1  7 wk p.i. | mouse | GC | captured GC volume (x 10^7^ µm^3^) | counted and estimated number of Tfh cells  (x 10^4^) | raw reads (x10^6^) | total TCRβ sequences (x10^6^)* | unique TCRβ clonotypes | number of Tfh-clonotypes subjected to analysis  >median ** |
|  | 1 | left | 4.32 | 3.67 | 1.9 | 0.67 | 2408 | 1204 |
|  |  | right | 4.23 | 3.59 | 2.4 | 0.2 | 1033 | 514 |
|  | 2 | left | 4.15 | 3.62 | 2.0 | 0.47 | 2060 | 1028 |
|  |  | right | 4.64 | 3.93 | 2.1 | 0.56 | 644 | 322 |
|  | 3 | left | 4.16 | 3.54 | 1.6 | 0.31 | 954 | 474 |
|  |  | right | 3.75 | 3.36 | 2.3 | 0.24 | 394 | 197 |
|  | mean ± SD |  | 4.21 ± 0.29 | 3.62 ± 0.19 | 2.05 ± 0.29 | 0.41 ± 0.19 | 1248.83 ± 804 | 623.17 ± 402 |

| Ag1  2/10 wk p.i. | mouse | GC | captured GC volume (x 10^7^ µm^3^) | counted and estimated number of Tfh cells  (x 10^4^) | raw reads (x10^6^) | total TCRβ sequences (x10^6^)* | unique TCRβ clonotypes | number of Tfh-clonotypes subjected to analysis  >median ** |
| --- | --- | --- | --- | --- | --- | --- | --- | --- |
|  | 1 | left  2 wk | 3.91 | 3.24 | 2.0 | 0.34 | 2219 | 1103 |
|  | 2 |  | 3.98 | 3.13 | 1.8 | 0.60 | 1375 | 686 |
|  | 3 |  | 3.99 | 3.00 | 1.9 | 1.78 | 4771 | 2378 |
|  | 4 |  | 4.04 | 3.30 | 2.0 | 0.91 | 723 | 361 |
|  | mean ± SD |  | 3.91 ± 0.05 | 3.17 ± 0.13 | 1.9 ± 0.1 | 0.91 ± 0.63 | 2272 ± 1775 | 1132 ± 884 |
|  | 1 | right  10 wk | 4.48 | 3.11 | 1.6 | 0.79 | 3821 | 1898 |
|  | 2 |  | 4.51 | 3.43 | 2.0 | 1.30 | 3680 | 1836 |
|  | 3 |  | 4.18 | 3.04 | 1.8 | 0.85 | 2064 | 1032 |
|  | 4 |  | 4.29 | 3.54 | 1.7 | 0.43 | 820 | 410 |
|  | mean ± SD |  | 4.37 ± 0.16 | 3.28 ± 0.24 | 1.78 ± 0.17 | 0.84 ± 0.36 | 2596 ± 1427 | 1294 ± 709 |

4-6 individual GC per pln were isolated by laser-microdissection and subjected to deep sequencing (Ag1 in SJL mice)

* TCRβ clonotype sequences that appeared only once were removed, ** only TCR sequences above the median were used for analysis
