# Supplementary material for: Receptor repertoires of murine follicular T helper cells reveal a high clonal overlap in separate lymph nodes in autoimmunity
Source: eLife. 2021 Aug 17;10:e70053. doi: 10.7554/eLife.70053 (PMC8370764; doi:10.7554/eLife.70053)
Supplement: Supplementary file 2. [file elife-70053-supp2.docx]

Supplementary file 2. Skin lesions: sizes, T cell numbers, raw reads, total and unique TCRβ sequences (Ag1/SJL)

| Ag1  4 wk p.i. | mouse | skin lesion | estimated size (x 10^7^ µm^3^) | counted and estimated number of T cells in skin lesions (x 10^4^) | raw reads (x10^6^) | total TCRβ sequences (x10^6^) | unique TCRβ clonotypes | number of Tfh-clonotypes subjected to analysis |
| --- | --- | --- | --- | --- | --- | --- | --- | --- |
|  | 1 | left | 123.0 | 69.2 | 0.6 | 0.17 | 1669 | 834 |
|  |  | right | 103.0 | 67.1 | 1.9 | 0.82 | 310 | 155 |
|  | 2 | left | 104.0 | 76.3 | 1.6 | 0.40 | 662 | 330 |
|  |  | right | 175.0 | 118.0 | 1.7 | 0.93 | 1263 | 626 |
|  | 3 | left | 199.0 | 150.0 | 1.6 | 0.44 | 670 | 335 |
|  |  | right | 134.0 | 123 | 1.9 | 0.86 | 840 | 420 |
|  | mean ± SD |  | 139.67 ± 39.23 | 100.60 ± 34.48 | 1.55 ± 0.48 | 0.60 ± 0.31 | 902.33 ± 486.69 | 450.00 ± 242.48 |

Skin lesions of ears were isolated and subjected to deep sequencing. * all sequences that appeared only once had been removed, data available at ^1^

1. Niebuhr, M. *et al.* Epidermal Damage Induces Th1 Polarization and Defines the Site of Inflammation in Murine Epidermolysis Bullosa Acquisita. *J Invest Dermatol* **140**, 1713-1722 e1719 (2020).
