# Supplementary material for: Receptor repertoires of murine follicular T helper cells reveal a high clonal overlap in separate lymph nodes in autoimmunity
Source: eLife. 2021 Aug 17;10:e70053. doi: 10.7554/eLife.70053 (PMC8370764; doi:10.7554/eLife.70053)
Supplement: Supplementary file 3. [file elife-70053-supp3.docx]

Supplementary file 3. Tfh isolated by flow cytometry, T cell numbers, raw reads, total and unique TCRβ sequences (Ag1/SJLH2s)

| Ag1  4 wks p.i. | mouse | Tfh  CD4+/PD1^high^/  CXCR5^high^ | counted and estimated number of Tfh cells  (x 10^4^) | raw reads (x10^6^) | total TCRβ sequences (x10^6^)* | unique TCRβ clonotypes | number of Tfh-clonotypes subjected to analysis  >median ** |
| --- | --- | --- | --- | --- | --- | --- | --- |
|  | 1 | left | 4.3 | 2.0 | 1.68 | 11522 | 5744 |
|  |  | right | 5.0 | 2.2 | 1.76 | 13418 | 6615 |
|  | 2 | left | 4.5 | 2.0 | 1.68 | 11942 | 5891 |
|  |  | right | 3.6 | 2.1 | 1.89 | 12988 | 6489 |
|  | 3 | left | 5.0 | 1.8 | 1.43 | 8420 | 4181 |
|  |  | right | 5.0 | 2.4 | 2.2 | 11579 | 5768 |
|  | mean ± SD |  | 4.57 ± 0.56 | 2.08 ± 0.2 | 1.77 ±0.26 | 11644 ± 1758,15 | 5781,33 ± 868,38 |

Tfh were isolated from left and right pln and subjected subjected to deep sequencing. * all sequences that appeared only once had been removed, ** only TCR sequences above the median were used for analysis
