# Supplementary material for: Receptor repertoires of murine follicular T helper cells reveal a high clonal overlap in separate lymph nodes in autoimmunity
Source: eLife. 2021 Aug 17;10:e70053. doi: 10.7554/eLife.70053 (PMC8370764; doi:10.7554/eLife.70053)
Supplement: Supplementary file 4. [file elife-70053-supp4.docx]

Supplementary file 4: Volumes, T cell numbers, raw reads, total and unique TCRβ sequences from laser-captured GC of pln (Ag2/C57BL6-H2s)

| PBS  5 wk p.i. | mouse | GC | captured GC volume (x 10^7^ µm^3^) | raw reads (x10^6^) | total TCRβ sequences (x10^6^) | unique TCRβ clonotypes | number of Tfh-clonotypes subjected to analysis  >median ** |
| --- | --- | --- | --- | --- | --- | --- | --- |
|  | 1 | left | 3.74 | 1.86 | 1.66 | 9338 | 4653 |
|  |  | right | 3.90 | 0.76 | 0.60 | 6951 | 3421 |
|  | 2 | left | 3.73 | 1.79 | 1.29 | 1652 | 826 |
|  |  | right | 3.79 | 0.72 | 0.50 | 4218 | 2087 |
|  | 3 | left | 3.81 | 1.53 | 1.31 | 3361 | 1680 |
|  |  | right | 3.19 | 0.73 | 0.59 | 2973 | 1478 |
|  | mean ± SD |  | 3.69 ± 0.16 | 1.23 ± 0.55 | 0.99 ± 0.5 | 4749 ± 2858 | 2357 ± 1418 |
| Ag2  5 wk p.i. | mouse | GC | captured GC volume (x 10^7^ µm^3^) | raw reads (x10^6^) | total TCRβ sequences (x10^6^) | unique TCRβ clonotypes | number of Tfh-clonotypes subjected to analysis |
|  | 1 | left | 3.66 | 1.83 | 1.16 | 1332 | 661 |
|  |  | right | 3.86 | 1.35 | 0.47 | 3514 | 1754 |
|  | 2 | left | 2.43 | 2.00 | 1.14 | 3758 | 1871 |
|  |  | right | 3.62 | 1.59 | 0.85 | 2495 | 1236 |
|  | 3 | left | 3.20 | 1.98 | 1.26 | 1452 | 726 |
|  |  | right | 3.88 | 1.33 | 1.10 | 3821 | 1896 |
|  | mean ± SD |  | 3.44 ± 0.41 | 1.68 ± 0.3 | 1.0 ± 0.29 | 2729 ± 1141 | 1357 ± 567 |

4-6 individual GC per pln were isolated by laser-microdissection and subjected to deep sequencing (Ag2/TM in C57BL6). * all sequences that appeared only once had been removed, ** only TCR sequences above the median were used for analysis
