# Supplementary material for: Receptor repertoires of murine follicular T helper cells reveal a high clonal overlap in separate lymph nodes in autoimmunity
Source: eLife. 2021 Aug 17;10:e70053. doi: 10.7554/eLife.70053 (PMC8370764; doi:10.7554/eLife.70053)
Supplement: Supplementary file 5. [file elife-70053-supp5.docx]

Supplementary file 5. entire pln: raw reads, total and unique TCRβ sequences (Ag1/SJLH2s)

| naive | mouse | pln | raw reads (x10^6^) | total TCRβ sequences (x10^6^)* | unique TCRβ clonotypes |
| --- | --- | --- | --- | --- | --- |
|  | 1 | left | 2.0 | 0.51 | 17300 |
|  |  | right | 1.6 | 1.28 | 75600 |
|  | 2 | left | 1.7 | 1.30 | 23900 |
|  |  | right | 1.5 | 1.24 | 32000 |
|  | 3 | left | 1.7 | 1.40 | 23200 |
|  |  | right | 1.5 | 1.52 | 81400 |
|  | mean ± SD |  | 1.67 ± 0.19 | 1.21 ± 0.36 | 42'233 ± 28538 |
| Ag1  1 days p.i. | mouse | pln | raw reads (x10^6^) | total TCRβ sequences (x10^6^) | unique TCRβ clonotypes |
|  | 1 | left | 1.2 | 1.13 | 104300 |
|  |  | right | 1.0 | 0.73 | 67400 |
|  | 2 | left | 1.0 | 0.81 | 72400 |
|  |  | right | 1.0 | 0.96 | 67400 |
|  | 3 | left | 1.1 | 1.05 | 50500 |
|  |  | right | 0.7 | 0.64 | 58000 |
|  | mean ± SD |  | 1.00 ± 0.17 | 0.89 ± 0.19 | 70'000 ± 18547 |
| Ag1  3 days p.i. | mouse | pln | raw reads (x10^6^) | total TCRβ sequences (x10^6^)* | unique TCRβ clonotypes |
|  | 1 | left | 1.8 | 1.68 | 112700 |
|  |  | right | 1.5 | 1.38 | 67200 |
|  | 2 | left | 1.2 | 1.16 | 76000 |
|  |  | right | 1.7 | 1.55 | 35500 |
|  | 3 | left | 1.3 | 1.25 | 53500 |
|  |  | right | 1.6 | 1.49 | 79300 |
|  | mean ± SD |  | 1.52 ± 0.23 | 1.42 ± 0.19 | 70'700 ± 26127 |

Left and right pln were isolated and subjected to deep sequencing. * all sequences that appeared only once had been removed
