# Supplementary material for: Receptor repertoires of murine follicular T helper cells reveal a high clonal overlap in separate lymph nodes in autoimmunity
Source: eLife. 2021 Aug 17;10:e70053. doi: 10.7554/eLife.70053 (PMC8370764; doi:10.7554/eLife.70053)
Supplement: Supplementary file 6. [file elife-70053-supp6.docx]

Supplementary File S6: Volumes, T cell numbers, raw reads, total and unique TCRβ sequences from

laser-captured GC of pln (GST/SJL-H2s)

| GST  4 wks p.i. | mouse | GC | captured GC volume (x 10^7^ µm^3^) | raw reads (x10^6^) | total TCRβ sequences (x10^6^) * | unique TCRβ clonotypes | number of Tfh-clonotypes subjected to analysis ** |
| --- | --- | --- | --- | --- | --- | --- | --- |
|  | 1 | left | 4.68 | 1.74 | 0.86 | 1348 | 674 |
|  |  | right | 4.22 | 1.70 | 0.61 | 439 | 218 |
|  | 2 | left | 2.88 | 1.87 | 1.24 | 1328 | 662 |
|  |  | right | 4.09 | 2.27 | 0.64 | 1974 | 981 |
|  | 3 | left | 4.68 | 1.59 | 0.45 | 2275 | 1136 |
|  |  | right | 3.99 | 1.71 | 1.03 | 1125 | 562 |
|  | mean ± SD |  | 4.09 ± 0.66 | 1.81 ± 0.24 | 0.81 ± 0.29 | 1414 ± 648 | 706 ± 323 |

4-6 individual GC per pln were isolated by laser-microdissection and subjected to deep sequencing (GST/SJL-H2s). * all sequences that appeared only once had been removed, ** only TCR sequences above the median were used for analysis
