# Supplementary material for: Receptor repertoires of murine follicular T helper cells reveal a high clonal overlap in separate lymph nodes in autoimmunity
Source: eLife. 2021 Aug 17;10:e70053. doi: 10.7554/eLife.70053 (PMC8370764; doi:10.7554/eLife.70053)
Supplement: Supplementary file 7. [file elife-70053-supp7.docx]

**Figure 4-figure supplementary file 2**

**Supplementary method**

Gene expression analysis

Five serial cryo-sections of pln were prepared for isolation of the total RNA using the innuPREP RNA Mini Kit (Analytik Jena, Hildesheim, Germany) and the gene expression was determined as previously described 20. Briefly, after reverse transcription, the cDNA and the respective primers were added to Taq Man qPCR Master Mix (Thermo Fisher Scientific, Waltham, USA) or SYBR Green qPCR Master Mix (Thermo Fisher Scientific, Waltham, USA) and amplified. All utilized primer (Biomers, Ulm, Germany) were designed to span at least an exon-exon junction and were titrated for optimal signal-to-noise ratio: Ki67 (for: 5’ATCATTGACCGCTCCTTTAGGT, rev: 5’GCTCGCCTTGATGGTTCCT), βActin (for: 5’GATGCTCCCCGGGCTGTATT, rev: 5’GGGGTACTTCAGGGTCAGGA), GAPDH (for: 5’GACGGCCGCATCTTCTTGT, rev: 5’CACACCGACCTTCACCATTTT, probe: 5’CAGTGCCAGCCTCGTCCCGTAGA), and MLN51 (for: 5′CCAAGCCAGCCTTCATTCTTG, rev: 5′TAACGCTTAGCTCGACCACTCTG, probe: 5′CACGGGAACTTCGAGGTGTGCCTAAC). For amplification and signal detection, the StepOne Plus Real-Time PCR (Thermo Fisher Scientific, Waltham, USA) was used. The relative gene expression was normalized to the geometric mean expression of the housekeeping genes βActin, GAPDH and MLN51 and is depicted as 2ΔΔct.
