# Supplementary material for: Receptor repertoires of murine follicular T helper cells reveal a high clonal overlap in separate lymph nodes in autoimmunity
Source: eLife. 2021 Aug 17;10:e70053. doi: 10.7554/eLife.70053 (PMC8370764; doi:10.7554/eLife.70053)
Supplement: Transparent reporting form [file elife-70053-transrepform.pdf.pdf]

Westbrook Centre
Milton Road
Cambridge CB4 1YG, UK
P 01223 855340
W elifesciences.org
T @elife

eLife's transparent reporting form

We encourage authors to provide detailed information *within their submission* to facilitate the interpretation and replication of experiments. Authors can upload supporting documentation to indicate the use of appropriate reporting guidelines for health-related research (see EQUATOR Network), life science research (see the BioSharing Information Resource), or the ARRIVE guidelines for reporting work involving animal research. Where applicable, authors should refer to any relevant reporting standards documents in this form.

If you have any questions, please consult our Journal Policies and/or contact us: editorial@elifesciences.org.

Sample-size estimation

- You should state whether an appropriate sample size was computed when the study was being designed
- You should state the statistical method of sample size computation and any required assumptions
- If no explicit power analysis was used, you should describe how you decided what sample (replicate) size (number) to use

Please outline where this information can be found within the submission (e.g., sections or figure legends), or explain why this information doesn't apply to your submission:

> 14 treated and mock-treated mice and two lymph nodes per mouse were isolated and used for TCR analysis. The observed effects were found with 2 antigens and two mouse strains and at two distinct time points. Due to the use of different time points, antigens and mouse strains, no explicit power analysis was used. Statistics was done with the non-parametric Mann-Whitney-U t-test. The number of mice and the statistical method used is found in the legend to each figure.

Replicates

- You should report how often each experiment was performed
- You should include a definition of biological versus technical replication
- The data obtained should be provided and sufficient information should be provided to indicate the number of independent biological and/or technical replicates
- If you encountered any outliers, you should describe how these were handled
- Criteria for exclusion/inclusion of data should be clearly stated
- High-throughput sequence data should be uploaded before submission, with a private link for reviewers provided (these are available from both GEO and ArrayExpress)

Please outline where this information can be found within the submission (e.g., sections or figure legends), or explain why this information doesn't apply to your submission:

> The robustness of the observed effects were reproduced as biological replications with different antigens, mouse strains, at two time points and with different T cell sampling methods. All data were included. Outliers were not encountered. High-throughput sequences of T cell receptors were deposited at the SRA: BioProject's metadata is available at https://dataview.ncbi.nlm.nih.gov/object/PRJNA731654?reviewer=5d7jabnjosp979hfdr2fnmlkhv. This information is found in figure legends and at the data availability section.

eLife Sciences Publications, Ltd is a limited liability non-profit non-stock corporation incorporated in the State of Delaware, USA, with company number 5030732, and is registered in the UK with company number FC030576 and branch number BR015634 at the address Westbrook Centre, Milton Road, Cambridge CB4 1YG.

Statistical reporting

- Statistical analysis methods should be described and justified
- Raw data should be presented in figures whenever informative to do so (typically when N per group is less than 10)
- For each experiment, you should identify the statistical tests used, exact values of N, definitions of center, methods of multiple test correction, and dispersion and precision measures (e.g., mean, median, SD, SEM, confidence intervals; and, for the major substantive results, a measure of effect size (e.g., Pearson's r, Cohen's d)
- Report exact p-values wherever possible alongside the summary statistics and 95% confidence intervals. These should be reported for all key questions and not only when the p-value is less than 0.05.

Please outline where this information can be found within the submission (e.g., sections or figure legends), or explain why this information doesn't apply to your submission:

> To compare more than two independent groups the nonparametric Kruskal-Wallis test was used (Fig. 1, figure 3-figure supplement 1). For comparison of two independent groups the Mann-Whitney U test (Fig. 2, Fig, 3a, Fig. 4) and for comparison of many variables between 2 groups, the 2way Anova with Sidaks correction test was used (Fig. 3b). Statistical analysis is found in the figure legends and in the Material and Method section.

(For large datasets, or papers with a very large number of statistical tests, you may upload a single table file with tests, Ns, etc., with reference to sections in the manuscript.)

Group allocation

- Indicate how samples were allocated into experimental groups (in the case of clinical studies, please specify allocation to treatment method); if randomization was used, please also state if restricted randomization was applied
- Indicate if masking was used during group allocation, data collection and/or data analysis

Please outline where this information can be found within the submission (e.g., sections or figure legends), or explain why this information doesn't apply to your submission:

> Mice were randomly allocated into the experimental groups and were randomly distributed in cages.

Additional data files ("source data")

- We encourage you to upload relevant additional data files, such as numerical data that are represented as a graph in a figure, or as a summary table
- Where provided, these should be in the most useful format, and they can be uploaded as "Source data" files linked to a main figure or table
- Include model definition files including the full list of parameters used
- Include code used for data analysis (e.g., R, MatLab)
- Avoid stating that data files are "available upon request"

Please indicate the figures or tables for which source data files have been provided:

> Raw data for high-throughput sequences of T cell receptors were deposited at the SRA: BioProject's metadata is available at https://dataview.ncbi.nlm.nih.gov/object/PRJNA731654?reviewer=5d7jabnjosp979hfdr2fnmlkhv. Movies for assessment of germinal center are uploaded. TCR analysis was performed with MiTCR and MiXCR (Figure 2-figure supplement 1). TCR packages or Immunarch was used for data extraction.

eLife Sciences Publications, Ltd is a limited liability non-profit non-stock corporation incorporated in the State of Delaware, USA, with company number 5030732, and is registered in the UK with company number FC030576 and branch number BR015634 at the address Westbrook Centre, Milton Road, Cambridge CB4 1YG.
